# Editorial: Male Idiopathic Infertility: Novel Possible Targets, Volume I

**DOI:** 10.3389/fendo.2021.797228

**Published:** 2021-11-17

**Authors:** Rossella Cannarella, Rosita A. Condorelli, Davor Jezek, Aldo E. Calogero

**Affiliations:** ^1^ Department of Clinical and Experimental Medicine, University of Catania, Catania, Italy; ^2^ Department of Transfusion Medicine and Transplantation Biology, University Hospital Zagreb, Zagreb, Croatia

**Keywords:** male infertility, spermatogenesis, proteomic, epigenetic, genetic

Male reproductive health represents an issue of increasing importance, especially considering that the total sperm count has halved is the last forty years (1). In Western countries, infertility affects about 15% of couples of childbearing ages (2). About half of these cases involve the male partner alone or is associated with a female factor of infertility (3). However, despite a thorough diagnostic work-up, the cause of infertility remains elusive in up to ∼70% of cases (4). Therefore, identifying novel possible targets of male idiopathic infertility is of paramount relevance to better understand its pathophysiological mechanisms and etiology. This, in turn, will possibly help in identifying targeted treatments in the near future.

Several recent lines of evidence suggest a role for the sperm transcriptome in human fertility. The review article by Pantos et al. timely describes the importance of the miRNA-34/449 for male infertility. The deregulation of this miRNA could lead to sperm aggregation and agglutination, abnormalities of sperm ciliogenesis in the efferent ducts, and defective reabsorption of the fluid present in the seminiferous tubules with a consequent increase in intratubular hydrostatic pressure. These mechanisms are compatible with the onset of obstructive infertility. Furthermore, it has been suggested that this miRNA is involved in the first stage of meiotic division. Hence, its downregulation could also lead to spermatogenic failure. All this results in a wide range of phenotypes ranging from oligozoospermia to azoospermia. However, further studies are still needed to confirm the role of miRNA-34/449 in male infertility.

The study by Hong et al. showed that piRNA levels in seminal plasma are lower in asthenozoospermic patients than in normozoospermic controls. The topic of the study is of great interest as previous evidence, mainly in animals, has shown a cross-talk between piRNAs in epididymal exosomes and spermatozoa. Particularly, piRNAs are distributed to spermatozoa in the caput of the epididymis, and their content in the exosomes of the cauda is significantly lower than that of the caput (5).

Human papillomavirus (HPV) is another under-diagnosed cause of male infertility, able to cause asthenozoospermia and negatively influence embryo growth. A deep and comprehensive review of the animal and human evidence is provided by Muscianisi et al. According to their data, patients with apparently idiopathic male infertility should also be evaluated for HPV infection. This may help to decrease the rate of idiopathic infertile male patients.

Obesity and metabolic impairment may represent another potential cause of apparently idiopathic male infertility. The meta-analysis by Zhao and Pang including 1731 patients have indeed shown that patients with metabolic syndrome (MetS) have statistically significantly lower sperm concentration, total sperm count, progressive motility, and sperm vitality, and increased percentage of spermatozoa with DNA fragmentation (SDF) and of the percentage of spermatozoa with low mitochondrial membrane potential. Insulin has been shown to influence Sertoli cell (SC) function and proliferation (6), and the action of endogenous follicle-stimulating hormone (FSH) is impaired in patients with MetS (7). These data may explain, at least partially, the findings by Zhao and Pang (Arato et al.).

Two articles of this special issue focused on porcine SCs. The first shows how diverse FSH molecules can impact SC function differently in terms of comparative proteomic analysis. Although no definitive conclusion can be drawn in humans, this finding may point toward a personalized therapeutic approach for the treatment of male infertility (Arato et al.). The second one focuses on the immunological properties of SCs. Accordingly, *in vitro* exposure to bacteria and lipopolysaccharides (LPS) resulted in a cell switch from the typical SC to an antigen-presenting cell (8).

Published studies support the concept that already in late adolescence (about 18 years), a proportion of boys ranging from 1:4 to 1:7 has testicular hypotrophy (8, 9). Hence, it would be essential to understand the pre-adolescent stimuli that are implicated in SC proliferation and, hence, contributing to reach an adequate testicular volume (TV). One of these stimuli is insulin-like growth factor 1 that has shown proliferative effects in porcine SCs *in vitro* (10). A retrospective analysis in patients with growth hormone deficiency (GHD) showed that, when treated with growth hormone (GH), patients had an increase of the TV over time as well as the age of puberty onset. Those who received treatment late had testicular hypotrophy and puberty delay (Cannarella et al.). These findings are in line with the effect of GH/IGF1 on testicular growth. Hypothetically, borderline-low GH-IGF1 serum levels in childhood and adolescence may be responsible for borderline-low testicular volumes in adulthood, although further evidence is needed to confirm this.

Next-generation sequencing (NGS) analyses represent a novel approach that may help to clarify the genetic etiology of oligozoospermia or azoospermia. Genetic panels for spermatogenetic failure have been developed to address this issue. As shown by the study of Precone et al., this panel could help to increase the diagnostic rate of male infertility.

Interestingly, oxidative stress (OS) is known as a cause of male infertility. Increased OS is indeed able to cause DNA double-strand breaks, in turn damaging sperm function. The review article by Aitken and Baker supports the concept that OS may damage susceptible regions of sperm DNA that encode for genes involved in male infertility, cancer, imprinted diseases, and behavioral disorders.

Abnormal sperm methylation has been observed in patients with infertility. On this account, the article by Franzago et al. evaluated the effect of a vegan diet on sperm epigenetics. Particularly, they found that a vegan diet can change the methylation rate of the *FTO* gene at the sperm level, which may impact transgenerational inheritance.

Lastly, the study by Li et al. evaluated seminal plasma proteomic profiles to understand any difference in patients with Kallmann syndrome compared to healthy men.

Taken together, the studies published in this Research Topic provide novel epigenetic, proteomic, transcriptomic targets of idiopathic male infertility, suggesting a unifying hypothesis and highlighting novel aspects of SC function. These data may help to reach a better comprehension of the causes of infertility. However, further efforts from the scientific community are still expected to understand the causes of idiopathic male infertility better.

## Author Contributions

RC and AEC conceived the study. RC wrote the original draft. AEC and DJ revised the draft and made revisions. RAC approved the manuscript. All authors contributed to the article and approved the submitted version.

## Conflict of Interest

The authors declare that the research was conducted in the absence of any commercial or financial relationships that could be construed as a potential conflict of interest.

## Publisher’s Note

All claims expressed in this article are solely those of the authors and do not necessarily represent those of their affiliated organizations, or those of the publisher, the editors and the reviewers. Any product that may be evaluated in this article, or claim that may be made by its manufacturer, is not guaranteed or endorsed by the publisher.
